# Aging in psoriasis vulgaris: female patients are epigenetically older than healthy controls

**DOI:** 10.1186/s12979-021-00220-5

**Published:** 2021-03-03

**Authors:** Pavel Borsky, Marcela Chmelarova, Zdenek Fiala, Kvetoslava Hamakova, Vladimir Palicka, Jan Krejsek, Ctirad Andrys, Jan Kremlacek, Vit Rehacek, Martin Beranek, Andrea Malkova, Tereza Svadlakova, Drahomira Holmannova, Lenka Borska

**Affiliations:** 1 Institute of Preventive Medicine, Faculty of Medicine in Hradec Kralove, Charles University, Simkova 870, 50038 Hradec Kralove, Czech Republic; 2 Institute of Clinical Biochemistry and Diagnostics, University Hospital Hradec Kralove and Faculty of Medicine in Hradec Kralove, Charles University, Hradec Kralove, Czech Republic; 3 Clinic of Dermatology and Venereology, University Hospital Hradec Kralove, Hradec Kralove, Czech Republic; 4 Institute of Clinical Immunology and Allergology, University Hospital and Faculty of Medicine in Hradec Kralove, Charles University, Hradec Kralove, Czech Republic; 5 Institute of Pathological Physiology, Faculty of Medicine in Hradec Kralove, Charles University, Hradec Kralove, Czech Republic; 6 Transfusion Center, University Hospital, 500 03 Hradec Kralove, Czech Republic

**Keywords:** Psoriasis, Aging, Epigenetic clock, Comorbidities

## Abstract

**Background:**

Psoriasis vulgaris is a skin autoimmune disease. Psoriatic patients have significantly lowered life expectancy and suffer from various comorbidities. The main goal of the study was to determine whether psoriatic patients experience accelerated aging. As accelerated aging might be the reason for the higher prevalence of comorbidities at lower chronological ages, we also wanted to investigate the relationship between aging and selected parameters of frequent psoriatic comorbidities - endocan, vascular endothelial growth factor and interleukin-17. Samples were obtained from 28 patients and 42 healthy controls. Epigenetic age measurement was based on the Horvath clock. The levels of endocan, vascular endothelial growth factor and interleukin-17 were analyzed using standardized ELISA methods.

**Results:**

The difference between the epigenetic age and the chronological age of each individual subject did not increase with the increasing chronological age of patients. We cannot conclude that psoriasis causes accelerated aging. However, the epigenetic and chronological age difference was significantly higher in female patients than in female controls, and the difference was correlated with endocan (r = 0.867, *p* = 0.0012) and vascular endothelial growth factor (r = 0.633, *p* = 0.0365) only in female patients.

**Conclusions:**

The findings suggest a possible presence of pathophysiological processes that occur only in female psoriatic patients. These processes make psoriatic females biologically older and might lead to an increased risk of comorbidity occurrence. This study also supports the idea that autoimmune diseases cause accelerated aging, which should be further explored in the future.

**Supplementary Information:**

The online version contains supplementary material available at 10.1186/s12979-021-00220-5.

## Introduction

Psoriasis is a multifactorial autoimmune disease with skin manifestations. It is associated with systemic inflammation. Increased levels of numerous cytokines, chemokines, growth factors and other molecules were found in the circulation of psoriatic patients [1–3]. The pathophysiology of psoriasis is very complex and consists of profound changes in innate and specific immunity [4]. The prevalence of the disease is 2–3% in the developed world and up to 8% in certain Nordic countries. The life expectancy of people with psoriasis is significantly lower than that of healthy controls [5]. Some studies suggest that psoriasis shortens the lifespan of patients by 4 years and maybe up to 10 years [6, 7]. Psoriatic patients commonly suffer from various comorbidities, such as cardiovascular diseases, diabetes, lymphoma or depression [5]. It is possible that the immune system of psoriatic patients is under immense stress due to the consistent pathological function of its components. Numerous studies have supported the conclusion that there is an imbalance between various compartments of the immune system [8–10]. This constant imbalance of homeostasis might give rise to a faster accumulation of damage, a phenomenon called accelerated aging.

Aging is characterized by a progressive decline in cellular function and organismal fitness and an increased risk of age-associated diseases and death [11]. Many theories of aging have emerged, yet the theory that seems to finally connect all the causes of aging is the theory of damage accumulation, which states that every metabolic process in an organism is the cause of a certain small degree of damage [12].. This theoretical assumption gave rise to a unified view on the changes during aging in the publication called Hallmarks of Aging. The article stated an interconnected list of 9 causes of aging, the types of damage that occur in the body: genomic instability, telomere attrition, epigenetic alterations, the loss of proteostasis, deregulated nutrient-sensing, mitochondrial dysfunction, cellular senescence, stem cell exhaustion and altered intercellular communication [13]. Considering this theory plausible means that certain conditions might cause more damage to the cells, tissues and organs of our body, therefore causing accelerated aging. The accelerated aging in this sense is the process of faster damage accumulation. We hypothesized that psoriatic patients might experience accelerated aging.

As mentioned above, epigenetic alterations represent one of the serious damage types in the body. Although genetics define the development and functional and morphological form of an organism, epigenetic mechanisms have an essential role in modulating attributes by regulating gene expression. These mechanisms allow the organism to adapt to stimuli and are therefore subjected to change over time [14]. Several studies have led to knowledge that certain parts of the genome change their methylation specifically throughout the aging process of the organism or tissue [15, 16]. The epigenetic clock method was first introduced by Steve Horvath and consists of measuring methylation percentages in specified CpG islands of the genome. These CpG islands change their methylation saturation throughout the lifetime of a person in a specific manner. Some of them lose methylation, and some of them become more methylated. Using artificial intelligence, it is possible to determine the chronological age of a healthy subject [16, 17]. Patients suffering from some diseases have been found to experience accelerated aging, meaning that their epigenetic clock measures older age than their true chronological age [18, 19]. A study by Marioni et al. found that the epigenetic clock correlates with physical fitness, suggesting that human epigenetic age (and its acceleration or deceleration) is susceptible to change throughout the lifespan [20].

Very little is currently known about the epigenetic age of psoriatic patients. A previous study of DNA methylation age in involved and uninvolved psoriatic skin tissue showed no significant difference [21]. For our study, we decided to use whole-blood samples instead of skin tissue because psoriasis is no longer considered a simple skin disease. It is a complex systemic autoimmune disease with skin manifestations [1–3]. According to the literature, immune system imbalance is the root cause of the disease [22]. As most of the genetic material in whole blood comes from white blood cells circulating throughout all body parts, we see whole-blood testing as a better indicator of the biological age of psoriatic patients than skin tissue [23].

As already mentioned, in addition to the immune system, psoriasis also affects other organs and systems, including the cardiovascular system. All these influences (inducing comorbidities) can accelerate the aging process [24–27]. Endocan (endothelial cell-specific molecule-1) is a molecule mainly expressed and secreted by endothelial cells. There is increasing evidence in recent literature that endocan is associated with atherosclerosis-related pathogenic processes, such as endothelial dysfunction, inflammation and angiogenesis, and therefore plays roles in cardiovascular disease [24]. Vascular endothelial growth factor (VEGF) is a potent stimulator of angiogenesis that is able to create a local pro-angiogenic environment by mobilizing endothelial progenitor cells [25]. Aggressive atherosclerotic plaque development and accelerated neovascularization of the vascular wall were seen following the administration of VEGF in laboratory experiments [28]. IL-17 is a cytokine expressed mainly by Th17 cells but also by others, such as CD8+ cells and natural killer T cells. It is a very important cytokine in the pathophysiology of psoriasis. Antibodies targeting IL-17 were found to be very effective as a treatment for moderate to severe plaque psoriasis. IL-17 is a critical mediator of inflammation due to its capacity to synergize with other inflammatory signals [27].

The main goal of our study was to determine whether psoriatic patients experience accelerated aging and to investigate the relationship between accelerated aging and parameters contributing to the higher prevalence of psoriatic comorbidities.

## Materials and methods

### Observed groups of subjects

The study group consisted of 28 patients (11 females and 17 males) with acute skin manifestations of psoriasis vulgaris. The median age was 47.2 years, range 20–65 years. Body mass index (BMI) was calculated as the ratio of weight to height squared (kg/m^2^). The acute intensity of psoriasis was calculated from basic characteristics of the disease status (desquamation, erythema and skin infiltration) and expressed as the PASI (Psoriasis Area and Severity Index) score [29]. The patients were without any immunomodulation therapy for at least 2 months before enrollment in the study. None of them had ever received systemic psoriasis therapy. Their exposure history was assessed using a questionnaire. Patients with acute infections, psoriatic arthritis, or other chronic inflammatory diseases were excluded. Sex, smoking habit, chronological age, BMI, PASI and disease onset of each individual patient is described int the Table 5 in supplementary. Six patients (3 men and 3 women) were diagnosed with comorbidities by the time of the sample collection and were taking proper medication for their disease: Diabetes mellitus II (*N* = 3; oral hypoglycemic), Hypertension (N = 3; ACE inhibitor or betablocker or calcium blocker), Dyslipidemia (*N* = 2; statin), Arrythmia (*N* = 1; no medication).

The control group consisted of 42 healthy blood donors (21 females and 21 males), whose median age was 51.6 years, range 20–65 years. Neither the patients nor the controls had been treated with any drugs influencing the inflammatory response for at least 2 weeks before the study. A sufficient power of the study was assured by sample size calculations before the enrollment of the study.

All the samples were collected throughout the time period of 2018 and 2019.

### Blood collection

Whole-blood samples treated with EDTA were collected and stored at − 70 °C until use. Blood serum was isolated by centrifugation for 10 min at 1300 g and stored at − 70 °C until analysis. Repeated thawing and freezing were avoided.

### Epigenetic clock analysis (epigenetic age)

Whole-blood samples treated with EDTA were protected by the DNA/RNA Shield™ reagent from Zymo Research Corp. (Irvine, CA 92614, USA., http://www.zymoresearch.com – Cat. No. R1100). Samples of DNA were purified from whole blood using the Quick-DNA™ Miniprep Plus kit (Zymo Research Corp., Cat. No. D4068). All the samples passed the quality control check performed by Nanodrop. Bisulfite conversion was performed using the EZ DNA Methylation-Lightning™ kit (Zymo Research Corp., Cat. No. D5030) according to the standard protocol. Samples were then enriched for sequencing of > 500 age-associated gene loci. Whole-blood sample DNA methylation values were obtained from the sequence data and used to assess DNA age according to the proprietary DNAge® predictor of Zymo Research.

### Endocan, VEGF and IL-17 analyses

The serum concentrations were analyzed using a Human ESM1 (Endocan) ELISA kit produced by Abcam, UK. Instructions from the manufacturer were always followed. Serum samples were diluted 10-fold, and the range of measurement was 300 to 20,000 pg/ml. Serum levels of VEGF were determined using ELISA with a commercial Quantikine ELISA Human VEGF kit (R&D Systems, MN, USA). Samples were not diluted, and the range of measurement was 15.6 to 1000 pg/ml. The assessment of IL-17 concentrations was performed using a Quantikine Human IL-17 kit from R&D Systems, MN, USA. Serum samples were not diluted, and the range of measurement was 20 to 2000 pg/ml.

### Statistical analysis

All data were statistically processed with Statistica version 13.5.0.17 (TIBCO Software Inc., Palo Alto, CA 94304 USA). Based on the Shapiro–Wilk test for the distribution of data, either the parametric or nonparametric test was used to ensure the proper test sensitivity. Associations between the parameters were evaluated by Pearson’s correlation test and Spearman’s rank correlation test. Intergroup differences were assessed using Student’s t-test or the Mann–Whitney U test. The differences were considered statistically significant when the probability level (p) was below the alpha level of 0.05.

### Age difference

By dividing the epigenetic age (EpiA) by the chronological age (ChronA) and subtracting the number 1 for each individual sample, we obtained a parameter that we call the age difference (Age diff). Its positive value means that the subject is epigenetically older than what their actual chronological age states. Age diff is the parameter showing whether each individual subject is older epigenetically than chronologically. The parameter is usually shown as a percentage.

## Results

The median PASI score in the group of patients was 15.6 (*N* = 28; interquartile range 12.3–26.3). The median age (ChronA) in the groups of patients, controls and subgroups of men and women is depicted on Table 1. was 47.2 (*N* = 28; interquartile range 39.4–53.5), and in the group of controls, the median age was 51.6 (*N* = 42; interquartile range 43.2–58.0). BMI was significantly elevated in the group of patients compared to the group of controls (patients, N = 28, median 28.3, interquartile range 24.7–30.5; controls, N = 42, median 26.2, interquartile range 23.6–28.7; *p* = 0.0398).

**Table 1:** Demographic data showing chronological age of groups. “N” stands for the number of subjects, “Q1” stands for first quartile, “Q3” stands for third quartile

	Controls				Patients				
	N	Median	Q1	Q3	N	Median	Q1	Q3	*p*-value
Men & Women	42	51.6	43.1	58.0	28	47.2	39.4	53.5	0.099
Men	21	51.9	50.7	58.2	17	47.9	41.4	52.9	0.089
Women	21	51.0	40.4	57.4	11	41.2	37.8	55.0	0.427

**Fig. 1:**
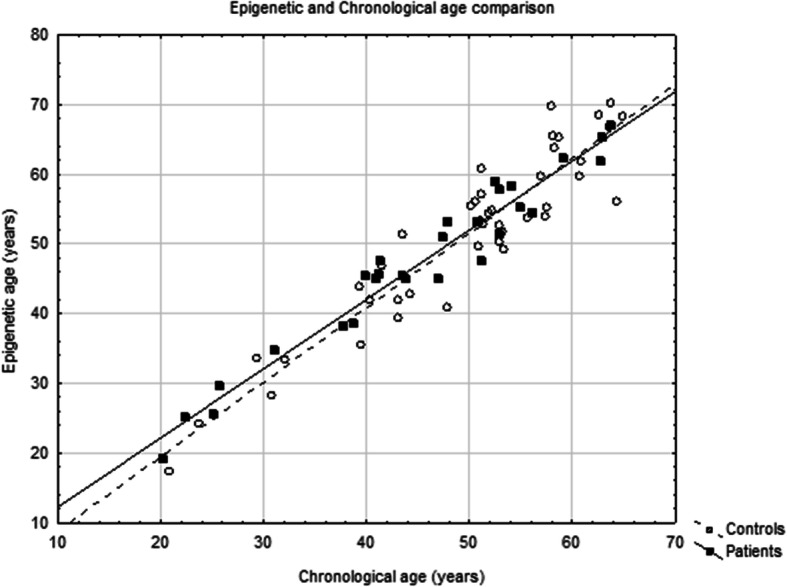
Scatterplot chart shows controls as white circles and patients as black squares

**Fig. 2:**
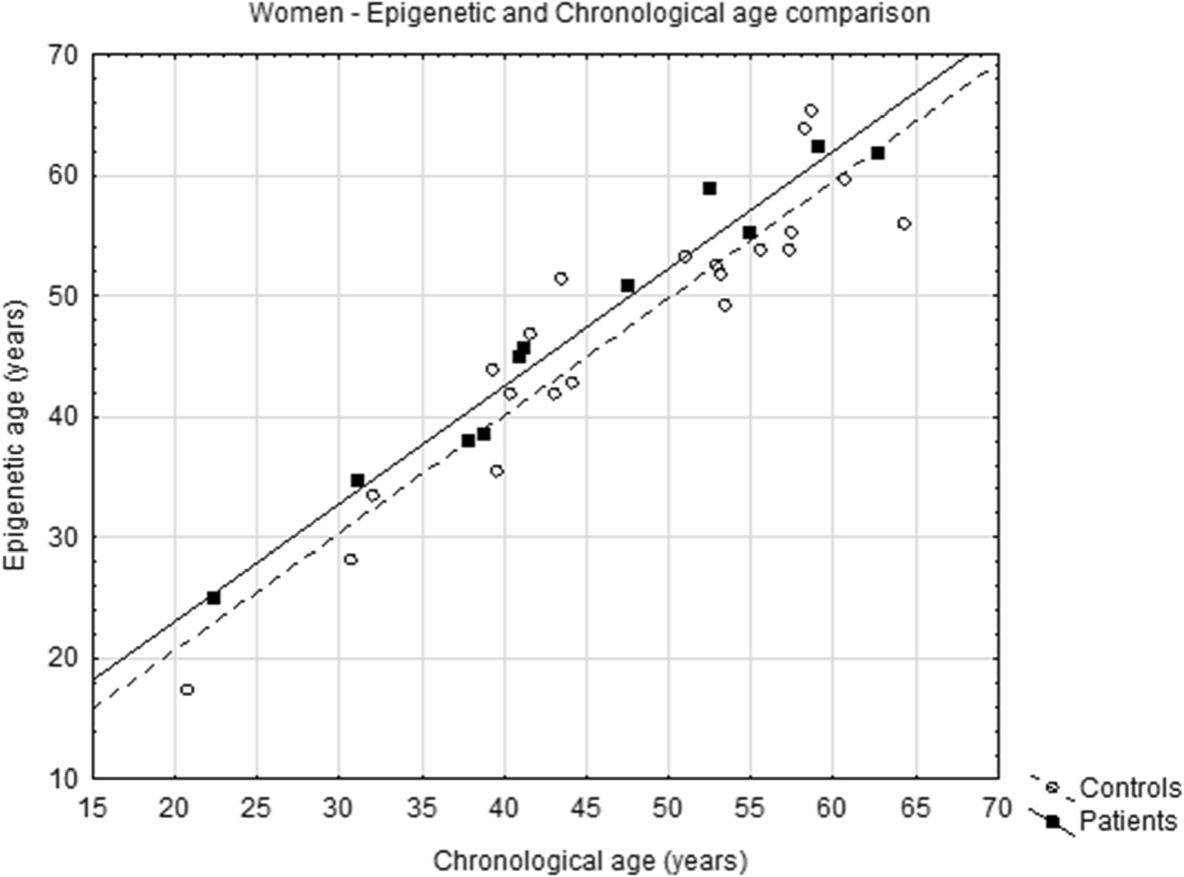
Scatterplot depicts the relationship of epigenetic and chronological age in women with psoriasis (patients, black squares) and in healthy women (controls, white circles)

**Fig. 3:**
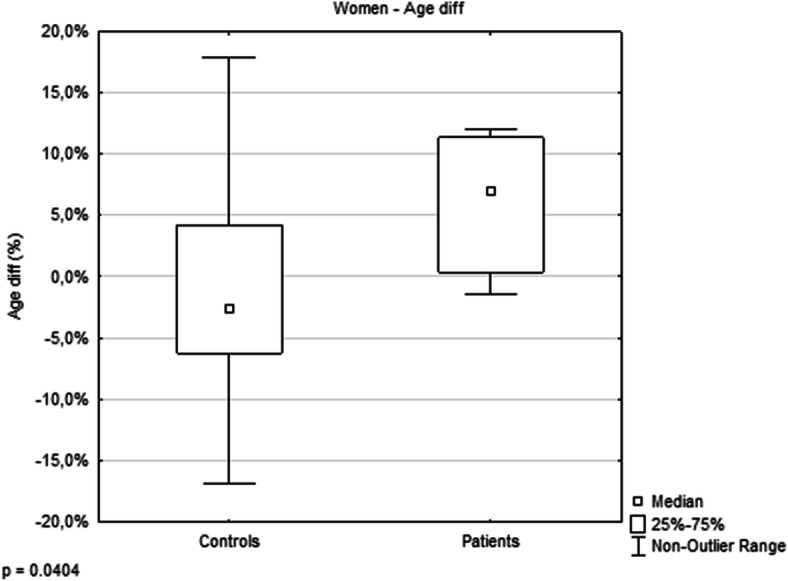
Women with psoriasis are epigenetically older than healthy women (*p* = 0.0404). An outlier is any data point value >75th percentile + 1.5*(75th percentile - 25th percentile) or any data point <25th percentile – 1.5*(75th percentile - 25th percentile)

**Fig. 4:**
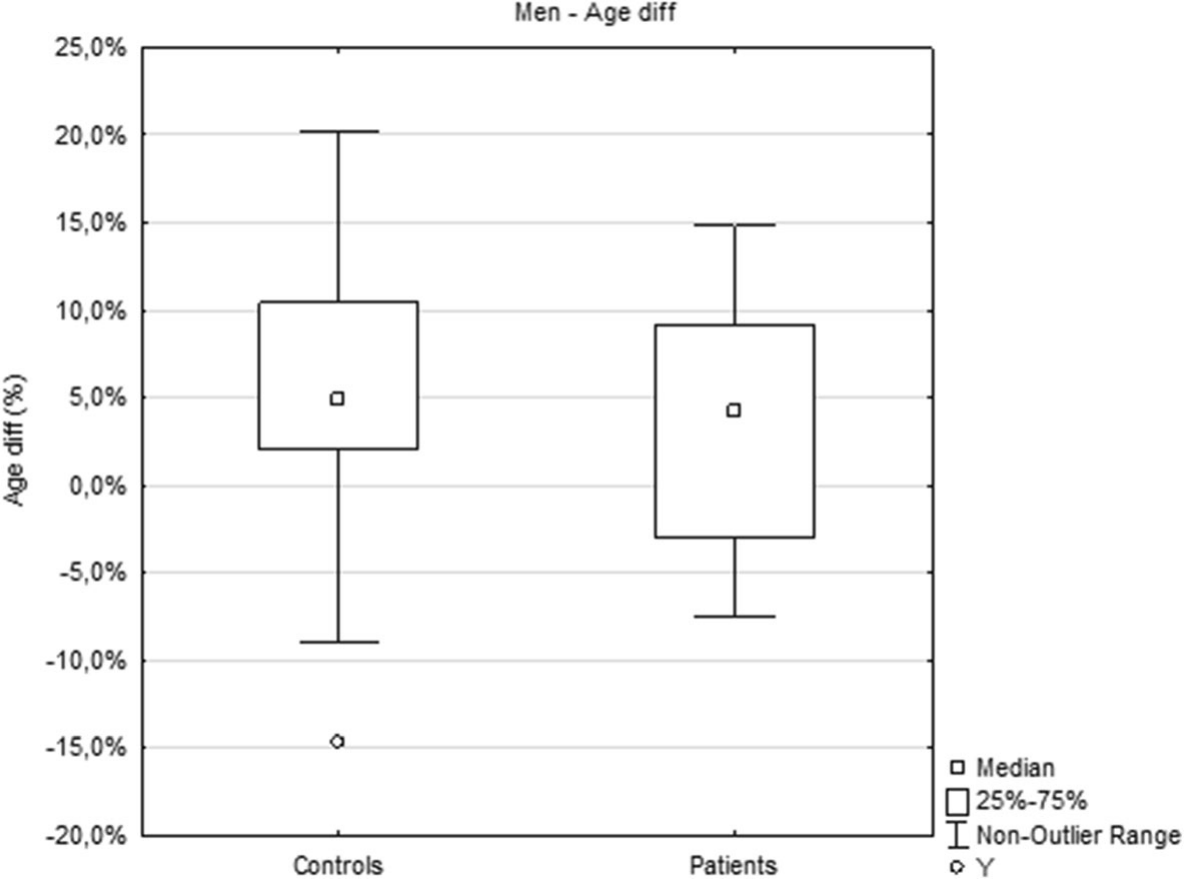
There is no statistically significant difference in the Age diff of men with psoriasis. Y is an outlier. An outlier is any data point value >75th percentile + 1.5*(75th percentile - 25th percentile) or any data point <25th percentile – 1.5*(75th percentile - 25th percentile)

The analysis of the relationship between epigenetic age and chronological age did not show a statistically significant difference between controls and patients (Fig. 1). However, the Age diff of each individual woman with psoriasis was found to be higher than the Age diff of healthy women (%; patients, *N* = 11, median 7.0, interquartile range 0.4–11.0; controls, *N* = 21, median − 3.0, interquartile range − 6.0–4.0; *p* = 0.0404; Fig. 2 and Fig. 3). In terms of years, the median difference between EpiA and ChronA was 3.2 in psoriatic women (interquartile range 0.2–4.1 years) compared to − 1.3 in healthy women (interquartile range − 2.6–2.2 years). There was no such relationship discovered in men with psoriasis (Fig. 4). In female controls, the median ChronA was 51.1 years (interquartile range 40.4–57.4), and in female patients, the median ChronA was 41.2 years (interquartile range 37.8–55.0).

We found no correlation between Age diff and the studied parameters (endocan, VEGF, IL-17) in either healthy men or patients. We found no correlation between Age diff and the studied parameters in healthy women. However, we found a significant relationship between Age diff and 2 studied parameters (endocan, VEGF) in women with psoriasis. The relationships are depicted in Table 2 (healthy controls) and Table 3 (patients). No correlation was found between Age diff and the disease duration (*N* = 28, median 8.5, interquartile range 4.5–22.5, years).

**Table 2:** Data from healthy controls showing relationships between Age diff and other parameters. “r” means Pearson’s or Spearman’s coefficient, “P” means Pearson’s correlation analysis was used, and “S” means Spearman’s correlation analysis was used. Statistically significant results are shown in bold

		Male and Female			Male			Female		
		r	p-value	S/P	r	p-value	S/P	r	p-value	S/P
Age diff	Endocan	0.014	0.945	S	−0.236	0.484	S	0.113	0.667	S
	VEGF	−0.129	0.512	S	−0.182	0.593	S	−0.169	0.516	S
	IL-17	−0.240	0.219	P	0.158	0.644	P	0.399	0.113	P
	BMI	0.063	0.693	S	−0.088	0.705	S	−0.036	0.876	S

**Table 3:** Data from patients showing relationships between Age diff and other parameters. “r” means Pearson’s or Spearman’s coefficient, “P” means Pearson’s correlation analysis was used, and “S” means Spearman’s correlation analysis was used. Statistically significant results are shown in bold

		Male and Female			Male			Female		
		r	p-value	S/P	r	p-value	S/P	r	*p*-value	S/P
Age diff	Endocan	0.365	0.067	S	0.034	0.901	S	**0.867**	**0.001**	S
	VEGF	0.064	0.746	S	−0.027	0.918	S	**0.633**	**0.036**	S
	IL-17	−0.101	0.609	P	−0.010	0.969	P	−0.450	0.165	P
	BMI	−0.252	0.196	S	−0.190	0.465	S	−0.518	0.102	S
	PASI	−0.267	0.169	S	−0.119	0.649	S	**−0.627**	**0.039**	S

In patients, we found significantly elevated levels of IL-17 (pg/ml; patients, N = 28, median 23.35, interquartile range 19.40–26.50; controls, *N* = 28, median 18.00, interquartile range 15.30–19.25; p < 0.0001) and significantly lower levels of endocan compared to controls (pg/ml; patients, *N* = 26, median 278, interquartile range 243–346; controls, N = 28, median 387, interquartile range 296–483; *p* = 0.0006). More samples were not measured due to technical issues.

Significantly lower levels of endocan and higher levels of IL-17 were also found in the subgroup of male patients: endocan (pg/ml; patients, *N* = 16, median 274, interquartile range 246–330; controls, *N* = 11, median 429, interquartile range 300–507; *p* = 0.0042; one more patient sample was not tested due to technical error), and IL-17 (pg/ml; patients, *N* = 17, median 22.30, interquartile range 18.90–25.10; controls, N = 11, median 18.90, interquartile range 15.30–21.30; *p* = 0.0286). Significantly higher levels of IL-17 were found in the subgroup of female patients (pg/ml; patients, N = 11, median 24.30, interquartile range 21.50–28.90; controls, N = 17, median 15.70, interquartile range 15.30–18.90; *p* < 0.0001).

Other significant correlations between the studied parameters in healthy subjects, psoriatic patients and subgroups of men and women are shown in Table 3 (controls) and Table 4 (patients) in the supplementary file. In healthy controls, these correlations are IL-17 and ChronA, IL-17 and BMI, and ChronA and BMI; in patients, these correlations are endocan and BMI, endocan and VEGF, and VEGF and PASI score.

## Discussion

The epigenetic clock method has been used to compare biological age in various studies. These studies compared the effects of race, sex, exercise, lifestyle factors and disease [16, 20, 30]. EpiA strictly correlated with ChronA in healthy people; however, it largely deviated in pathological conditions. The most prominent example is Down syndrome, which was proven to accelerate the epigenetic aging of patients’ whole blood. The study demonstrated that trisomy 21 significantly increases the age of whole blood and brain tissue (on average by 6.6 years, *p* = 7.0 × 10^− 14^) [19]. In our study, we were not been able to conclude that there was a pathophysiological condition causing accelerated aging in the whole blood of our patients. Their Age diff was not increasing within their lifetime, within their exposure to the disease (Fig. 1).

On the other hand, we found that women with psoriasis were epigenetically older than healthy women (Fig. 2). This does not apply to men, although it is usually men who have higher epigenetic aging rates than women (in blood) [30]. In our study, we could not state that there was accelerated aging in women with psoriasis, as the difference between EpiA and ChronA did not progress with increasing ChronA. The correct description is that women with psoriasis were epigenetically older by, on average, 2.5 years (6.5%) than healthy women. This finding might explain why the lifespan of women with psoriasis is shorter. A recent meta-analysis indicated that each 5-year increase in epigenetic age was associated with an 8 to 15% increased risk of mortality [31].

If our conclusions are correct, why is this phenomenon present only in women, not men? It appears that there must be sex-specific metabolic pathways that are altered by psoriasis because only women with psoriasis were epigenetically older than healthy controls. It seems that there is sex specificity in terms of blood composition [32], the immune system [33], hormones, lifestyle [34], the microbiome [35] and many others.

Differences in immune functions and responses contribute to health and lifespan disparities between sexes. Women have stronger immune responses to infections and vaccination than men [33, 36]. Paradoxically, the stronger immune response comes at a price, which is the higher incidence of autoimmune diseases in women [36]. This relationship has not yet been clearly explained. Sex hormones contribute to the development and activity of the immune system, accounting for differences in sex-related immune responses. Both the innate immune system and the adaptive immune systems bear receptors for sex hormones and respond to hormonal cues [33, 36].

Yamagata et al. showed in their study that DNA methyltransferase 3a and 3b (DNMT3a and DNMT3b) expression in endometrial stromal cells is downregulated by medroxyprogesterone acetate and estrogen [37]. It was also reported that DNA methylation status can be altered by a variety of factors, including steroids and vitamins [38]. On the other hand, DNA methylation affects estrogen receptors in female reproductive organs [39, 40]. These findings suggest a close relationship between DNA methylation and female sex steroid hormones. Further studies must clarify the molecular mechanisms and potential correlations of steroid hormones and blood methylation profiles, but this seems a likely path for future studies.

Psoriasis, an autoimmune disease, is affected by sex specificity. However, studies focusing on the prevalence of psoriasis by sex have not come to similar conclusions. Higher prevalence was described in women in the USA and Norway, and lower prevalence was found in Denmark and Australia [41, 42]. Many factors can exacerbate psoriasis, including hormone fluctuations and stress. In recent studies, women even report higher levels of stigmatization (a strong predictor of quality of life) due to the presence of the disease [43]. A study from 2016 stated that psoriasis risk appeared to be higher in women with irregular menstrual cycles in adulthood [44].

In our study, we found that there was no correlation in healthy men or women between Age diff and endocan or VEGF (Tab. 1). There was also no correlation between these parameters in psoriatic men. However, we found significant relationships in women with psoriasis. Even though the number of subjects in our study is relatively low, this fact further supports the validity of the sex-specific results of our EpiA analysis and the fact that psoriasis combined with female sex could be a cause of profound metabolic changes.

Endocan and VEGF play roles in the pathogenesis of endothelial dysfunction and atherosclerosis [24, 28]. Our results could support the hypothesis that there is an association between psoriasis and epigenetic age, atherosclerosis, cardiovascular comorbidities, and shorter lifespan in women. The study of Garshick et al. clearly states that psoriasis is associated more strongly with cardiovascular disease in female patients than in male patients [45]. The conclusions of this study based on a group of young hospitalized patients (age ≥ 20 and < 35 years) are concordant with our results, i.e., that EpiA is constantly higher than ChronA throughout the lifetime of female patients, even from early ages. Thus, these findings could at least partially explain the reasons for the shorter lifespan of psoriatic women. Therefore, using treatment attacking the fundamental causes of aging (rejuvenation therapies or geroprotectors) might lead to a better outlook for psoriatic patients.

In our study, we found lower levels of endocan in psoriatic patients (*p* < 0.001). The difference was statistically significant in men; lower levels were also seen in women, but the difference was not significant (*p* = 0.08). The literature is inconsistent in its view on endocan in psoriasis. Some studies showed higher levels of endocan in patients [46, 47], whereas others measured lower levels in patients than in healthy controls [48]. Future research is needed to further clarify these findings.

IL-17 was significantly elevated in both men and women with psoriasis compared to the control group, which is in agreement with the available literature [49], yet we found no correlation between Age diff and IL-17. Although future research is needed, our preliminary data suggest that IL-17 and its related immune pathways would not cause higher EpiA in women with psoriasis.

Our study found a significant negative correlation between endocan and BMI in psoriatic women (r = − 0.661; *p* = 0.0376; Tab. 4), which corresponds to the literature [50, 51]. In women, endocan was significantly correlated with VEGF (r = 0.681; *p* = 0.0302; Tab. 4). A similar correlation between them was previously found, for example, in the vitreous fluid of diabetic retinopathy patients [52]; however, this has never been observed in psoriasis patients. Further, VEGF and PASI score had a negative relationship in female patients (r = − 0.661; *p* = 0.0269; Tab. 4), which means that acute severity of the disease might cause a decrease in VEGF. This is in contradiction to the findings of Zablotna et al., who found a positive correlation between serum VEGF and PASI score in patients of both sexes [53]. A possible explanation is the different proportion of sexes in the studies (50% in their study vs. 39% in ours) or the lower mean PASI score in their study (14.5 vs. 19.2). However, this finding seems strange given the scientific literature, which mostly describes a positive relationship between the PASI score and VEGF due to the overexpression of VEGF in psoriatic lesions [54, 55].

The PASI score was found to negatively correlate with Age diff in women (r = − 0.627; *p* = 0.0388; Tab. 2). Supposedly the acute severity of psoriasis (objectified by PASI score) does not reflect the changes of epigenome. We state the hypothesis that the acute symptoms of psoriasis might even be a repair reaction of the body. It is possible that the body of patients protects itself from the negative epigenetic changes in the long term by acute flares of the disease. A study by Jain et al. suggested that white blood cells change their epigenome quickly to better suit the threat they are facing [56]. However possible, the explanation is purely speculative with current state of knowledge. The result is unprecedented and will need more studies to be clarified.

It must be stated that the patients had overall higher BMI. Scientific literature describes that patients with psoriasis suffer from metabolic syndrome and overweight more frequently [57–59]. It is impossible to determine, whether the higher BMI contributed to the older epigenetic age of women with psoriasis, however, it seems unlikely due to negative non-significant relationship between Age diff and BMI in the subgroup.

In summary, our findings preliminary suggest that women with psoriasis are prone to changes in their epigenome that are associated with older biological age. It is currently impossible to determine whether the reason is genetic background or the specific stimulation of the immune system right after the onset of the disease or other factors. The shorter lifespan of men with psoriasis cannot be explained by higher epigenetic age. The question of whether their shorter lifespan is associated with accelerated aging at all or whether other hallmarks of aging are affected needs to be answered in the future.

Several limitations of the study should be mentioned. When generalizing the study conclusions, we must take into account (a) the relatively low number of subjects from only one geographical area, (b) the fact that only one hallmark of aging was analyzed (c) the use of blood – the heterogeneity of aging of different tissues might cause disparate results depending on type of samples used for the analysis, (d) the impossibility of comorbidities exclusion in psoriatic patients. The results must be further supported by more studies to be able to draw definite conclusions.

## Conclusions

We cannot generally declare that having psoriatic disease causes more damage and, therefore, accelerated aging over time. Age diff did not increase within the lifetime of patients. On the other hand, we found that Age diff was significantly higher in female patients than in healthy female controls, which suggests a possible presence of pathophysiological processes appearing only (or to larger extent) in female psoriatic patients (larger risk of comorbidities in psoriatic women). This finding is further supported by the fact that the correlations between Age diff and endocan or VEGF were found only in women with psoriasis.

The present study supports future research on aging in psoriasis and possible other autoimmune diseases.

## Supplementary Information

**Additional file 1.**

## Data Availability

The data used to support the findings of this study are available from the corresponding author upon request.

## Abbreviations

Age diff: Age difference
BMI: Body mass index
ChronA: Chronological age
DNMT3a: DNA methyltransferase 3a
DNMT3b: DNA methyltransferase 3b
EpiA: Epigenetic age
IL-17: Interleukin 17
PASI: Psoriasis area and severity index
VEGF: Vascular endothelial growth factor
