# Supplementary material for: Aging in psoriasis vulgaris: female patients are epigenetically older than healthy controls
Source: Immun Ageing. 2021 Mar 3;18:10. doi: 10.1186/s12979-021-00220-5 (PMC7927262; doi:10.1186/s12979-021-00220-5)
Supplement: Supplementary file 1 — Additional file 1. [file 12979_2021_220_MOESM1_ESM.docx]

|  | | | Male and Female | | Male | | Female | |
| --- | --- | --- | --- | --- | --- | --- | --- | --- |
|  |  |  | r | Sign. | r | Sign. | r | Sign. |
| Endocan | & | BMI | 0.158 | 0.423 | 0.018 | 0.958 | 0.355 | 0.162 |
| Endocan | & | VEGF | -0.122 | 0.536 | -0.064 | 0.853 | -0.154 | 0.554 |
| VEGF | & | PASI | - | - | - | - | - | - |
| IL-17 | & | ChronA | -0.299 | 0.122 | **-0.680** | **0.021** | -0.121 | 0.645 |
| IL-17 | & | BMI | -0.302 | 0.118 | **-0.900** | **0.000** | 0.033 | 0.899 |
| ChronA | & | BMI | **0.341** | **0.027** | **0.452** | **0.040** | 0.294 | 0.197 |

Table 3: Healthy controls, Spearman

|  | | | Male and Female | | Male | | Female | |
| --- | --- | --- | --- | --- | --- | --- | --- | --- |
|  |  |  | r | Sign. | r | Sign. | r | Sign. |
| Endocan | & | BMI | **-0.426** | **0.030** | -0.181 | 0.503 | **-0.661** | **0.038** |
| Endocan | & | VEGF | **0.448** | **0.022** | 0.398 | 0.127 | **0.681** | **0.030** |
| VEGF | & | PASI | **-0.467** | **0.012** | -0.476 | 0.054 | **-0.661** | **0.027** |
| IL-17 | & | ChronA | 0.214 | 0.275 | 0.163 | 0.532 | 0.418 | 0.201 |
| IL-17 | & | BMI | -0.019 | 0.923 | -0.272 | 0.291 | 0.382 | 0.247 |
| ChronA | & | BMI | 0.147 | 0.455 | 0.092 | 0.725 | 0.182 | 0.593 |

Table 4: Patients, Spearman

| Sex | Smoking | ChronA | BMI | PASI | Onset |
| --- | --- | --- | --- | --- | --- |
| Male | 0 | 20.3 | 28.0 | 27.0 | 2012 |
| Female | 0 | 22.4 | 20.5 | 15.6 | 2010 |
| Male | 1 | 25.1 | 24.7 | 6.4 | 2017 |
| Male | 1 | 25.8 | 23.5 | 13.7 | 2018 |
| Female | 0 | 31.2 | 28.3 | 19.7 | 2012 |
| Female | 1 | 37.8 | 32.2 | 16.2 | 2018 |
| Female | 0 | 38.7 | 44.7 | 37.6 | 1998 |
| Male | 0 | 40.0 | 30.5 | 19.8 | 2018 |
| Female | 0 | 40.9 | 24.6 | 12.0 | 2011 |
| Female | 1 | 41.2 | 28.2 | 13.4 | 1992 |
| Male | 1 | 41.4 | 29.5 | 8.8 | 1998 |
| Male | 1 | 43.6 | 24.5 | 13.7 | 2013 |
| Male | 1 | 43.9 | 30.3 | 8.0 | 2010 |
| Male | 1 | 46.9 | 27.4 | 62.1 | 1996 |
| Female | 1 | 47.5 | 24.8 | 20.3 | 2019 |
| Male | 1 | 47.9 | 30.6 | 27.0 | 2002 |
| Male | 0 | 50.8 | 26.1 | 11.2 | 2004 |
| Male | 1 | 51.3 | 32.2 | 11.6 | 2013 |
| Female | 0 | 52.5 | 30.3 | 6.8 | 1972 |
| Male | 1 | 52.9 | 24.3 | 12.6 | 1981 |
| Male | 0 | 52.9 | 24.3 | 13.8 | 1996 |
| Male | 1 | 54.1 | 30.5 | 43.2 | 2019 |
| Female | 0 | 55.0 | 42.4 | 15.0 | 2015 |
| Male | 1 | 56.1 | 36.9 | 40.5 | 1976 |
| Female | 0 | 59.1 | 27.5 | 25.5 | 2014 |
| Female | 0 | 62.7 | 29.3 | 21.8 | 1997 |
| Male | 0 | 62.9 | 30.0 | 15.6 | 2011 |
| Male | 1 | 63.8 | 21.8 | 34.0 | 1985 |

Table 5: Sex, smoking habit, chronological age, BMI, PASI and disease onset in the patients’ group
